# Supplementary material for: Polyvalent Bacterial Lysate with Potential Use to Treatment and Control of Recurrent Urinary Tract Infections
Source: Int J Mol Sci. 2024 Jun 3;25(11):6157. doi: 10.3390/ijms25116157 (PMC11173243; doi:10.3390/ijms25116157)
Supplement: Supplementary file 1 [file ijms-25-06157-s001.zip › ijms-3015770-supplementary.pdf]

Table S1. Primers used in the different PCR assays for the characterization of *Escherichia coli* strains associated with urinary tract infections

Target	Primer	Size (pb)	Reference
**Fimbrial and afimbrial adhesins**			
afa	GGCAGAGGGCCGGCAACAGGC<br>CCCGTAACGCGCCAGCATCTC	593	[63]
papG alelo II	GGGATGAGCGGGCCTTTGAT<br>CGGGCCCCCAAGTAACTCG	190	[63]
papG alelo III	GGCCTGCAATGGATTTACCTGG<br>CCACCAAATGACCATGCCAGAC	258	[63]
focG	CAGCACAGGCAGTGGATACGA<br>GAATGTCGCCTGCCCATTGCT	362	[63]
**Toxins**			
cnf-1	GCAGAACGACGTTCTTCATAAGTATC<br>ATCTTATACTGGATGGGATCATCTTGG	972	[64]
vat	AACGGTTGGTGGCAACAATCC<br>AGCCCTGTAGAATGGCGAGTA	421	[65]
**Regulators, serines and mycelaneans**			
set1A	TCACGCTACCATCAAGGA<br>TATCCCCCTTTGGCGGTA	309	[66]
**Biofilm formation**			
fluB (ag43)	AGGCAGGAGGAACTGCCAGT<br>TAAATGAGGGTGTCCCGTGCC	440	[10]
fluA (ag43)	CAGCCGGATCTGCGGCACT<br>ACTCTGGTGTTTCTGGCTGTT	340	[10]

Table S2. Conditions used for the detection of virulence- and phylogroup-associated genes in UPEC strains.

Initial denaturation	Denaturation	Alignment 30 s	Target						Extension	Final extension
		50 ºC	*iucD*	*vat*	*iss*					
		51 ºC	*pic*							
		52 ºC	*ompT*	*iutA*	*fyuA*	*feoB*				
		53 ºC	*irp2*	*hylD*	*cnf1*	*ompA*	*sit*			
		54 ºC	*papA*	*papC*	*ireA*					
96 ºC 2 min	95 ºC 30 s	56 ºC	*focG*						72 30 s	72 ºC 5 min
		57 ºC	*ibeA*	*kpsMT1*	*papG*	*iha*	*traT*	*fluA*		
		58 ºC	*ironD*	*tsh*						
		59 ºC	*mailX PAI*							
		61 ºC	*fluB*							
		63 ºC	*fimH*							

Table S3. Susceptibility and resistance profile of the UPEC strains that make up the UNAM-HIMFG lysate

***E. coli**	**AMP**	**PRL**	**MEL**	**AMC**	**TZP**	**KZ**	**MA**	**FEP**	**CFP**	**FOX**	**CRO**	**CAZ**	**CXM**	**MEM**	**F**	**ATM**	**CN**	**KA**	**K**	**TOB**	**S**	**TE**	**OF**	**CIP**	**NOR**	**NA**	**SXT**	**S3**	**W**	**C**	**FOS**
O25	R	R	R	R	R	R	R	R	R	S	R	R	R	R	R	R	R	R	R	R	R	R	R	R	R	R	S	R	S	S	R
O75	R	R	R	R	S	R	R	S	S	S	S	S	R	R	S	S	S	S	R	S	R	R	R	R	R	R	R	R	R	S	S
O9	R	R	R	R	R	R	R	R	R	R	R	R	R	R	R	R	R	R	R	R	R	S	R	R	R	R	R	R	R	R	S
O17	R	R	R	S	S	R	R	S	R	S	R	S	R	S	S	R	S	S	S	S	S	S	S	S	S	S	S	R	S	S	S
O20	R	R	R	R	R	R	R	R	R	R	R	R	R	R	R	S	S	R	R	R	R	R	R	R	R	R	R	R	R	R	R
O6	R	S	R	S	S	R	S	R	S	R	R	R	R	S	R	S	S	R	R	S	R	R	R	S	S	S	S	R	S	S	R
O8	R	S	S	R	S	R	S	S	S	S	R	S	R	S	S	S	S	S	S	S	S	S	S	S	S	S	S	R	S	S	S
O1	R	S	R	R	S	R	S	S	S	S	S	S	R	S	S	S	S	R	R	S	R	S	S	S	S	S	S	R	S	S	S
O16	R	R	R	R	R	R	R	R	R	S	R	R	R	S	S	R	S	S	S	R	R	R	S	R	S	R	R	R	R	S	S
O7	R	S	R	S	R	R	S	R	S	S	R	R	R	R	S	S	S	S	S	R	R	S	S	S	S	R	S	R	S	S	R

(AMP) ampicilin, (PRL) piperacilin, (MEL) mecilinam, (AMC) amoxicillin with clavulanic acid, (TZP) piperacillin with tazobactam (100 µg/10µg), cefozolin (30µ g), cefamandole (30µg), cefepime (30, (KZ) cefozolina, (MA) cefamandole, (FEP) cefepime, (CFP) cefoperazone, (FOX) cefoxitin, (CRO) ceftriaxone, (CAZ) ceftazidime, (CXM) cefuroxime, (MEM) meropenem, (F) nitrofurantoin, (ATM) aztreonam, (CN) gentamicin , (KA) amikacin, (K) kanamycin, (TOB) trobramycin , (S) streptomycin, (TE) tetraciclyne, (OF) ofloxacin, (CIP) ciprofloxacin, (NOR) norfloxacin, (NA) nalidixic acid, (STX) trimethoprim with sulfamethoxazole, (S3) sulfonamides, (W) trimethoprim , (C) chloramphenicol, (FOS) fosfomycin. (R) Resistant (S) Susceptible, (I) Intermediate, (DDS) dose-dependent susceptibility.
**Escherichia coli* serogroups

Table S4. Susceptibility and resistance of other urinary pathogens that make up the UNAM-HIMFG lysate

BAC	AMP	PRL	MEL	AMC	TZP	KZ	MA	FEP	CFP	FOX	CRO	CAZ	CXM	MEM	NF	ATM	CN	AK	K	TOB	S	TE	OF	CIP	NOR	NA	SXT	S3	W	C	FOS	VA
CF	ND	R	R	ND	R	ND	R	R	R	ND	R	R	ND	R	R	R	S	S	R	S	R	S	R	R	S	R	R	R	R	R	R	ND
KP	ND	R	R	S	R	R	S	S	S	R	R	R	R	R	R	S	S	S	R	S	R	S	S	R	S	R	R	R	S	R	R	ND
EF	S	S	R	S	S	ND	ND	ND	ND	ND	ND	ND	ND	R	S	ND	ND	ND	ND	ND	ND	R	R	R	S	ND	ND	ND	ND	R	S	S
SH	R	R	R	S	R	R	R	R	R	S	R	R	R	S	R	ND	S	S	S	S	S	R	S	S	S	ND	R	R	R	R	R	ND
KA	ND	R	R	ND	R	ND	R	R	S	ND	R	R	R	R	R	S	S	R	R	R	R	R	S	S	R	R	S	R	R	S	R	ND
PM	S	S	R	S	S	R	R	R	S	R	R	R	R	R	ND	R	S	R	R	S	R	ND	S	S	S	R	R	R	R	R	R	ND

(AMP) ampicilin, (PRL) piperacilin, (MEL) mecilinam, (AMC) amoxicillin with clavulanic acid, (TZP) piperacillin with tazobactam (100 µg/10◎g), cefozolin (30◎ g), cefamandole (30◎g), cefepime (30, (KZ) cefozolina, (MA) cefamandole, (FEP) cefepime, (CFP) cefoperazone, (FOX) cefoxitin, (CRO) ceftriaxone, (CAZ) ceftazidime, (CXM) cefuroxime, (MEM) meropenem, (NF) nitrofurantoin, (ATM) aztreonam, (CN) gentamicin , (AK) amikacin, (K) kanamycin, (TOB) trobramycin , (S) streptomycin, (TE) tetraciclyne, (OF) ofloxacin, (CIP) ciprofloxacin, (NOR) norfloxacin, (NA) nalidixic acid, (STX) trimethoprim with sulfamethoxazole, (S3) sulfonamides, (W) trimethoprim , (C) chloramphenicol, (FOS) fosfomycin. (VA) vancomycin. Abbreviations: (R) Resistant (S) Susceptible, (I) Intermediate, (DDS) dose-dependent susceptibility., (ND) Not determined by the presence of intrinsic resistance. BAC: Bacteria; CF: *Citrobacter freundii*; KP: *Klebsiella pneumoniae*; EF: *Enterococcus faecalis*; SH: *Stahpylococcus haemolyticus* ; KA: *Klebsiella aerogenes*; PM: *Proteus mirabilis*

Table S5 Virulence genes identified in *E. coli* strains that make up the UNAM-HIMFG lysate

**E. coli Strain serogroup*	P	**Fimbrial and afimbrial adhesins**								**Siderophores**							**Toxins**								**Others & Biofilm formation**					
			fimH	*sfa*	*iha*	*papA*	*papC*	*papG II*	*focG*	*iutA*	*iucD*	*feoB*	*fyuA*	*ireA*	*Irp-2*	*iroN*	*cnf-1*	*Set1*	*hylD*	*vat*	*sat*	*sitA*	*tratA*	*kpsMT (K1)*	*ompA*	*ompT*	*fluA*	*fluB*	*pic*	*malX (PAI)*
O25	B2		*fimH*		*iha*	*papA*	*papC*			*iutA*	*iucD*	*feoB*	*fyuA*		*irp-2*		*cnf-1*		*hlyD*		*sat*	*sitA*	*traT*		*ompA*		*fluA*			*malX (PAI)*
O75	B2									*iutA*	*iucD*	*feoB*			*irp-2*					*vat*	*sat*	*sitA*		*kpsMT (K1)*	*ompA*					
O9	A											*feoB*			*irp-2*															
O17	D		*fimH*									*feoB*			*irp-2*							*sitA*		*kpsMT (K1)*	*ompA*					
O20	A		*fimH*							*iutA*	*iucD*	*feoB*	*fyuA*		*irp-2*				*hylD*			*sitA*			*ompA*					
O6	B2		*fimH*	*sfa*					*focG*			*feoB*	*fyuA*		*irp-2*	*iroN*	*cnf-1*	*set1*	*hylD*	*vat*		*sitA*	*traT*	*kpsMT (K1)*	*ompA*			*fluB*	*pic*	
O8	B1		*fimH*									*feoB*													*ompA*					*malX (PAI)*
O1	B2		*fimH*			*papA*	*papC*	*papG II*		*iutA*	*iucD*	*feoB*	*fyuA*	*ireA*	*irp-2*	*iroN*			*hylD*	*vat*	*sat*	*sitA*	*traT*	*kpsMT (K1)*	*ompA*	*ompT*				*malX (PAI)*
O16	B2	*afa*	*fimH*									*feoB*			*irp-2*							*sitA*	*traT*		*ompA*	*ompT*				
O7	D		*fimH*				*papC*	*papG II*			*iucD*	*feoB*	*fyuA*		*irp-2*						*sat*	*sitA*		*kpsMT (K1)*	*ompA*	*ompT*				

Abbreviation: P: Phylogroup;
